# Facile Synthesis of 2D/2D Ti_2_C_3_/ZnIn_2_S_4_ Heterostructure for Enhanced Photocatalytic Hydrogen Generation

**DOI:** 10.3390/ijms24043936

**Published:** 2023-02-15

**Authors:** Yongjuan Chen, Yanfang Ge, Chunling Wu, Hua Tang, Xiu Luo, Jiao He, Liang Jiang, Zhiying Yan, Jiaqiang Wang

**Affiliations:** School of Chemical Sciences & Technology, Yunnan Province Engineering Research Center of Photocatalytic Treatment of Industrial Wastewater, School of Materials and Energy, Yunnan University, Kunming 650091, China

**Keywords:** ZnIn_2_S_4_, MXene, Ti_2_C_3_, photocatalytic, hydrogen generation

## Abstract

ZnIn_2_S_4_, a novel two-dimensional visible light-responsive photocatalyst, has attracted much attention in the photocatalytic evolution of H_2_ under visible light irradiation due to its attractive intrinsic photoelectric properties and geometric configuration. However, ZnIn_2_S_4_ still has severe charge recombination, which results in moderate photocatalytic performance. Herein, we report the successful synthesis of 2D/2D ZnIn_2_S_4_/Ti_3_C_2_ nanocomposites by a facile one-step hydrothermal method. The efficiency of the nanocomposites in photocatalytic hydrogen evolution under visible light irradiation was also evaluated for different ratios of Ti_3_C_2_, and the optimal photocatalytic activity was achieved at 5% Ti_3_C_2_. Importantly, the activity was significantly higher than that of pure ZnIn_2_S_4_, ZnIn_2_S_4_/Pt, and ZnIn_2_S_4_/graphene. The enhanced photocatalytic activity is mainly due to the close interfacial contact between Ti_3_C_2_ and ZnIn_2_S_4_ nanosheets, which amplifies the transport of photogenerated electrons and enhances the separation of photogenerated carriers. This research describes a novel approach for the synthesis of 2D MXenes for photocatalytic hydrogen production and expands the utility of MXene composite materials in the fields of energy storage and conversion.

## 1. Introduction

As a kind of sustainable clean energy, hydrogen energy has great application potential in solving the problem of energy shortage. Fujishima and Honda were the first to split water into H_2_ and O_2_ via a titania (TiO_2_) electrode in 1972 [1]. Since then, semiconductor photocatalytic hydrogen production has been purposefully pursued due to its potential applications in the production of clean hydrogen energy [2,3]. However, the application of photocatalysis is limited by the absorption of sunlight, the efficiency of charge separation, and the interfacial catalytic reaction [4].

One strategy to improve the photocatalytic activity is through the development and use of two-dimensional (2D) nanocrystallization of semiconductor photocatalytic materials. These materials offer larger specific surface area with more active sites, while the ultra-thin nano layer can shorten the carrier transport distance and facilitate its migration to the catalyst surface. ZnIn_2_S_4_ is a promising photocatalytic H_2_ generation photocatalyst due to its desirable band gap in the visible light region [5,6]. In general, ZnIn_2_S_4_ shows two different crystal phases of hexagonal lattice and cubic lattice. A large number of previous reports indicated that hexagonal phases had better stability and higher photocatalytic activity. Most often, flake structures have been observed in the morphology of hexagonal ZnIn_2_S_4_ due to the stacking of S-Zn-S-In-S-In-S layers [7,8,9,10]. Advantageously, the unique structure of 2D ZnIn_2_S_4_ nanosheets decreases the diffusion distance of electrons and improves the separation of photoinduced charges, while a large number of exposed surface atoms can provide rich active sites [11].

However, due to the low separation efficiency and migration capacity of photoexcited charge carriers, the photocatalytic activity of pure ZnIn_2_S_4_ is insufficient [12,13]. Over the years, numerous efforts have been made to improve ZnIn_2_S_4_, such as doping with metal ions [14,15], combination with semiconductor [16], construction of heterojunction [17], coupling cocatalyst [18], etc. The construction of 2D/2D heterostructures can form more charge transfer channels, which is conducive to the spatial separation and migration of carriers [19,20]. Therefore, the assembly of new heterostructures is considered an effective strategy to improve the photocatalytic performance of ZnIn_2_S_4_.

Ti_3_C_2_ MXene, as a new 2D material, has attracted extensive attention in photocatalysis because of its special metallic conductivity. Ti_3_C_2_ is rich in hydrophilic functional groups (-OH, -O, -F) on the surface, which is favorable for composites with semiconductor photocatalytic materials. A sufficiently positive Fermi level (0.71 eV vs. NHE) of Ti_3_C_2_ is conducive to the transfer of photoelectrons from semiconductors to Ti_3_C_2_, as well as the effective separation of electron–hole pairs. Recently, it has been reported that ZnIn_2_S_4_ nanosheets were grown in situ on Ti_3_C_2_ nanosheets to form a sandwich-like hierarchical heterostructure [21]. The unique hierarchical structure confers the system with superior photoexcited charge transport ability, which further accelerates photoexcited charge separation.

In this study, 2D/2D ZnIn_2_S_4_/Ti_3_C_2_ nanocomposites were successfully prepared by a simple one-step hydrothermal method, and the photocatalytic performance of the composites with different ratios of Ti_3_C_2_ was evaluated in the visible light region. The photocatalytic activity reached the maximum for a mass ratio of 5% Ti_3_C_2_, and it was significantly higher than that of pure ZnIn_2_S_4_, ZnIn_2_S_4_/Pt, and ZnIn_2_S_4_/graphene. The enhanced photocatalytic activity is mainly due to the close interfacial contact between Ti_3_C_2_ and ZnIn_2_S_4_ nanosheets, which accelerates the transport of photoelectrons and increases the separation of photogenerated carriers.

## 2. Results

### 2.1. Characterizations

**Figure 1:**
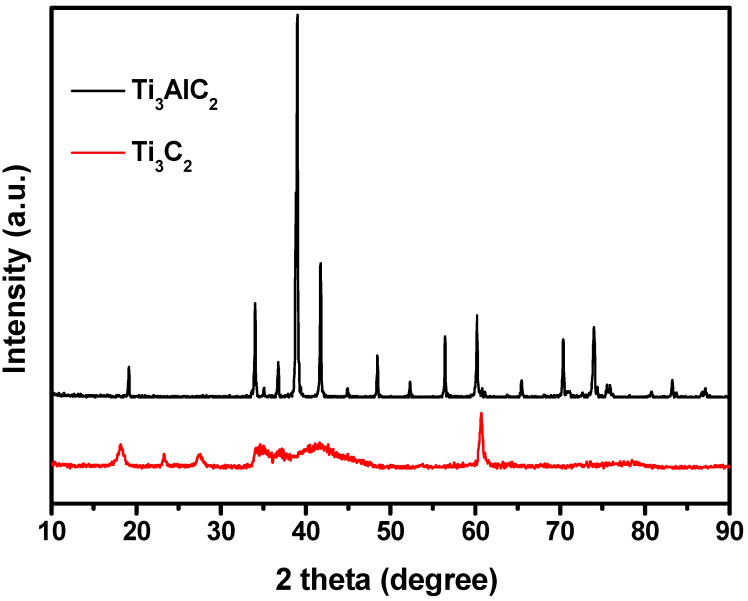
XRD patterns for Ti_3_AlC_2_ and obtained Ti_3_C_2_ by etching the Al layer of Ti_3_AlC_2_ with HF solution.

**Figure 2:**
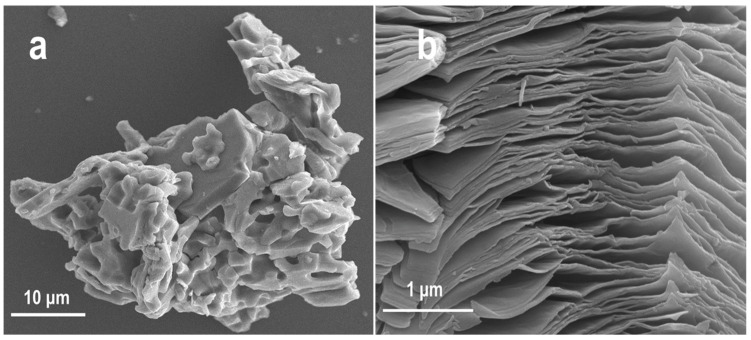
SEM images of (**a**) Ti_3_AlC_2_ and (**b**) obtained Ti_3_C_2_ by etching the Al layer of Ti_3_AlC_2_ with HF solution.

XRD was used to determine the crystalline phases of the samples, as shown in Figure 1. Notably, the largest diffraction peak for the (104) plane of Ti_3_AlC_2_, which was at 39.0°, disappeared after HF treatment, indicating the removal of Al layers of Ti_3_AlC_2_ [22]. In addition, the diffraction peaks of the (002) plane at 9.4° and the (004) plane at 19.1° shifted to lower degrees and become broader, which was ascribed to increase in the interlayer spacing of Ti_3_C_2_ [23]. This can also be evidenced by SEM images of Ti_3_C_2_ (Figure 2b). Compared with Ti_3_AlC_2_ (Figure 2a), Ti_3_C_2_ has an obviously layered microstructure. These results confirmed the successful transformation of Ti_3_AlC_2_ into layered Ti_3_C_2_.

**Figure 3:**
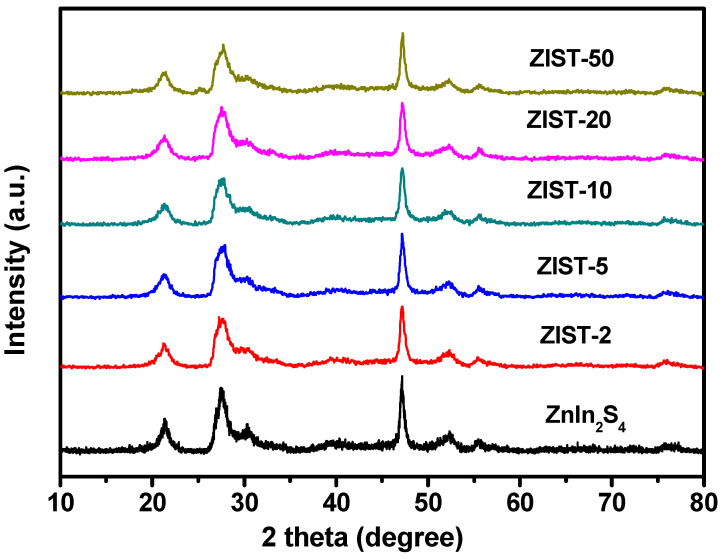
XRD patterns of ZnIn_2_S_4_/Ti_3_C_2_ composites with different Ti_3_C_2_ content.

ZnIn_2_S_4_/Ti_3_C_2_ nanocomposites were synthesized via a facile one-step hydrothermal method. The mass ratio of ZnIn_2_S_4_ to Ti_3_C_2_ was varied to determine the optimal composition, and mass ratios of 2 wt.%, 5 wt.%, 10 wt.%, 20 wt.%, and 50 wt.% corresponded to samples ZIST-2, ZIST-5, ZIST-10, ZIST-20, and ZIST-50, respectively. Figure 3 presents the XRD patterns of ZnIn_2_S_4_/Ti_3_C_2_ composites with different Ti_3_C_2_ content, and the ZnIn_2_S_4_/Ti_3_C_2_ composites had similar diffraction peaks to that of hexagonal ZnIn_2_S_4_ [18]. In addition, no obvious diffraction peaks of Ti_3_C_2_ were found in the XRD patterns of the composites, indicating the limited amount and weak diffraction intensity of Ti_3_C_2_. Meanwhile, no diffraction peak of TiO_2_ was found, which suggested Ti_3_C_2_ will not transform into TiO_2_ at 150 °C. Therefore, the temperature for sample synthesis is feasible in this work.

**Figure 4:**
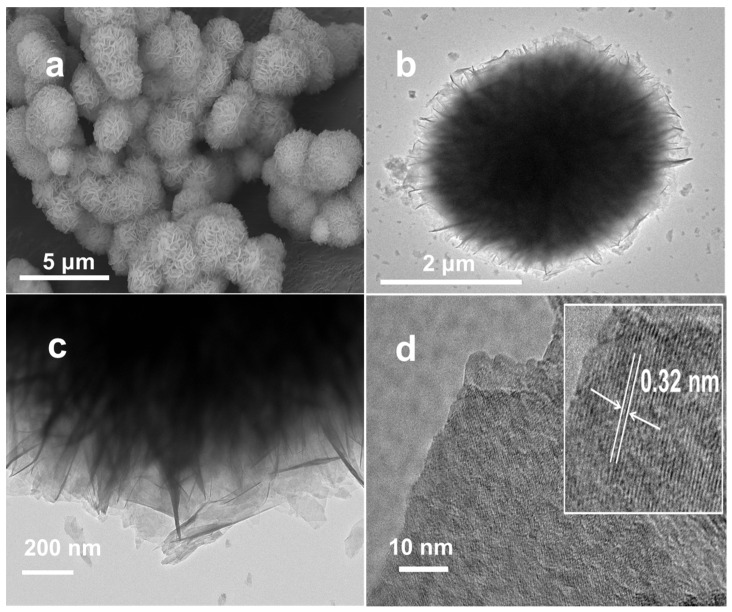
(**a**) Typical SEM and (**b**,**c**) TEM images of ZnIn_2_S_4_ at different magnifications, (**d**) and HRTEM image of ZnIn_2_S_4_ nanocrystallinity.

**Figure 5:**
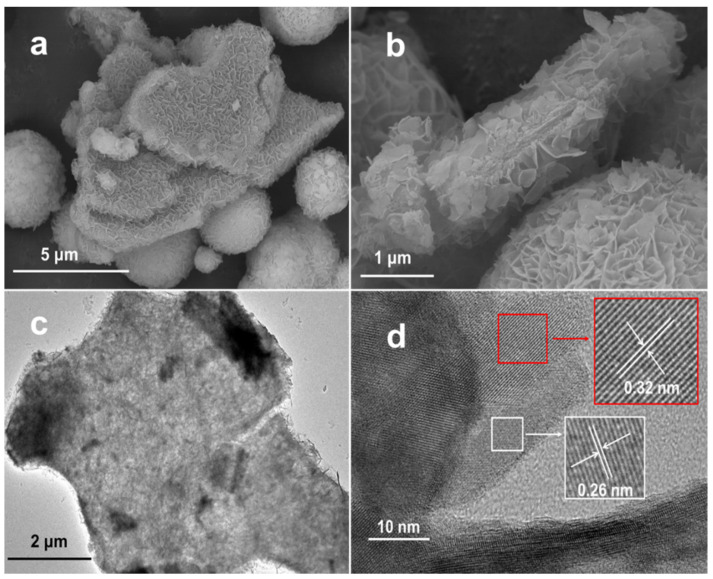
SEM images (**a**,**b**), (**c**) TEM, and (**d**) HRTEM images of ZnIn_2_S_4_/Ti_3_C_2_ composites (ZIST-5).

SEM and TEM were used to observe the morphologies of the samples. The pure ZnIn_2_S_4_ had a 3D hierarchical flower-like structure self-assembled by 2D nanosheets (Figure 4) according to previous reports [24,25]. The SEM images of ZnIn_2_S_4_/Ti_3_C_2_ composites (ZIST-5) showed ultrathin nanosheets of ZnIn_2_S_4_ densely populating the surface of the layered Ti_3_C_2_ (Figure 5a). It can be clearly observed that ZnIn_2_S_4_ is an outer layer closely attached to the of surface Ti_3_C_2_, which is the inner layer in Figure 5b. Ti_3_C_2_ retains an integrated lamellar structure in the TEM images (Figure 5c) of ZnIn_2_S_4_/Ti_3_C_2_. Lattice spacing of 0.32 nm and lattice fringes of 0.26 nm were determined in the HRTEM image of ZnIn_2_S_4_/Ti_3_C_2_, corresponding to the (102) plane of hexagonal ZnIn_2_S_4_ [18] and the (010) plane of Ti_3_C_2_, respectively [26]. These results indicate that ZnIn_2_S_4_ nanosheets were anchored on Ti_3_C_2_ rather than simply mixed.

**Figure 6:**
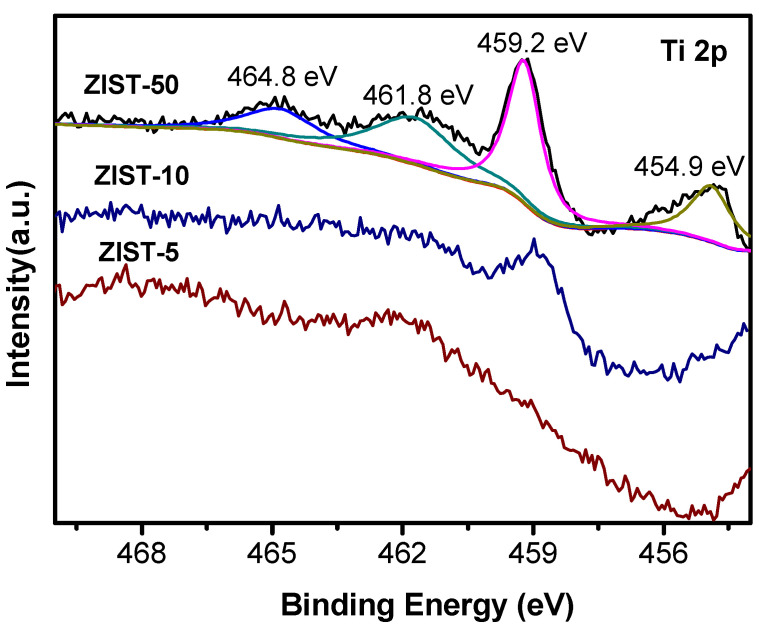
XPS Ti 2p spectra of ZnIn_2_S_4_/Ti_3_C_2_ composites with different Ti_3_C_2_ content.

**Figure 7:**
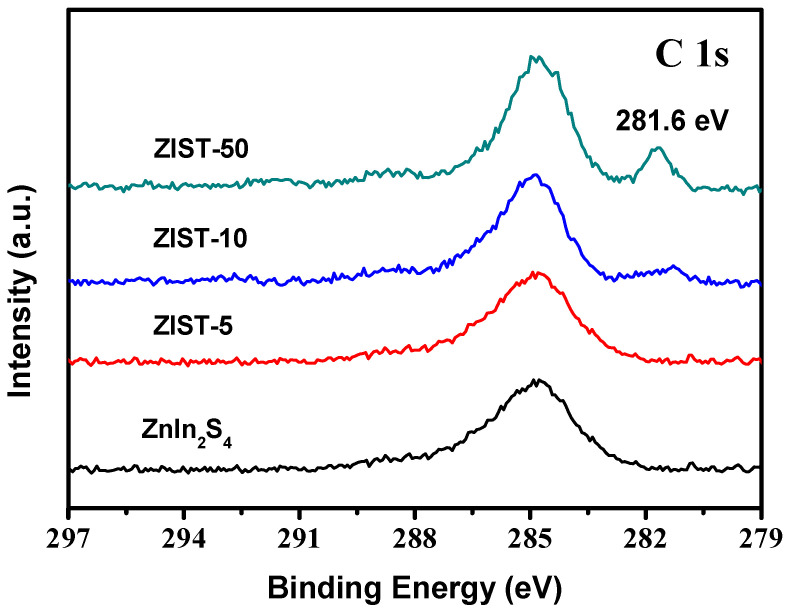
XPS C 1s spectra of ZnIn_2_S_4_ and ZnIn_2_S_4_/Ti_3_C_2_ composites with different Ti_3_C_2_ content.

**Figure 8:**
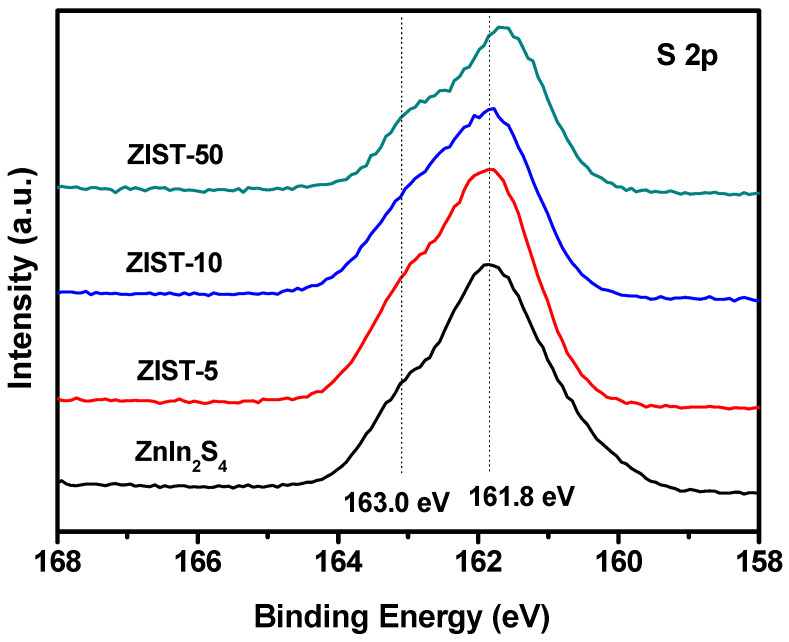
XPS S 2p spectra of ZnIn_2_S_4_ and ZnIn_2_S_4_/Ti_3_C_2_ composites with different Ti_3_C_2_ content.

**Figure 9:**
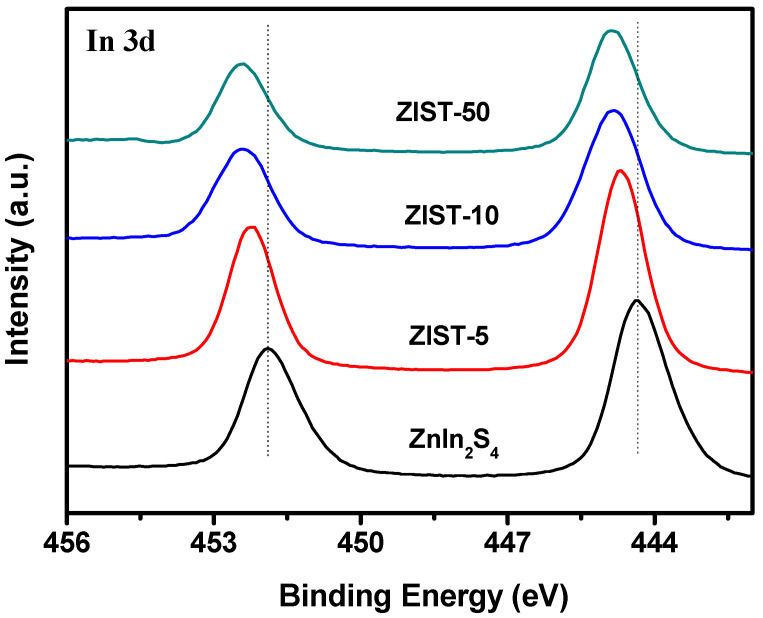
XPS In 3d spectra of ZnIn_2_S_4_ and ZnIn_2_S_4_/Ti_3_C_2_ composites with different Ti_3_C_2_ content.

**Figure 10:**
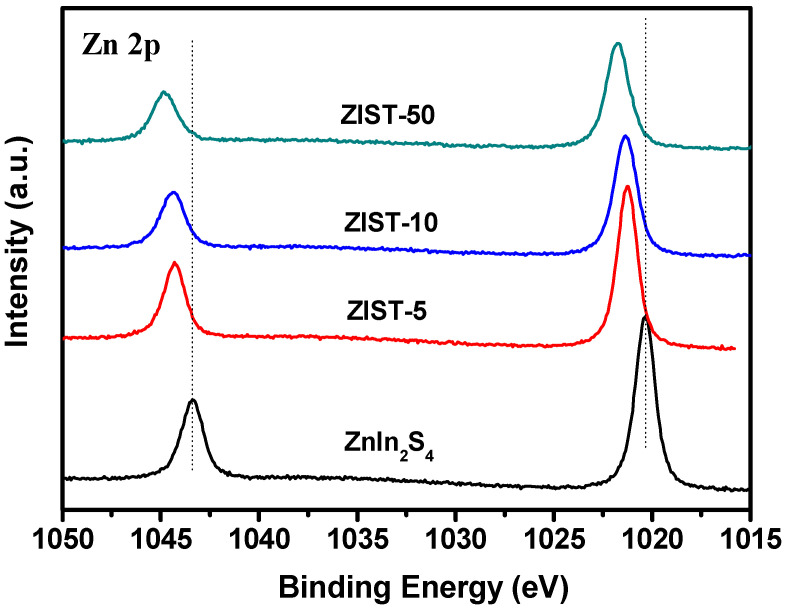
XPS Zn 2p spectra of ZnIn_2_S_4_ and ZnIn_2_S_4_/Ti_3_C_2_ composites with different Ti_3_C_2_ content.

Next, the surface chemistry of the photocatalyst was investigated by XPS. As shown in Figure 6, a weak signal was observed in the Ti 2p spectrum of ZIST-5, which is mainly due to the existence of Ti_3_C_2_ in the inner layer of the sample and its content is too low. With the increase in Ti_3_C_2_ content, the Ti 2p signal becomes stronger. When the mass ratio of Ti_3_C_2_ reaches 50% of ZnIn_2_S_4_ (ZIST-50), four peaks at 454.9, 459.2, 461.8, and 464.8 eV were observed in the Ti 2p spectrum, correlating with Ti-O 2p1/2, Ti-C 2p1/2, Ti-O 2p3/2, and Ti-C 2p3/2, respectively [27]. Ti-O is unlikely to be attributable to TiO_2_, because it can be seen from XRD that Ti_3_C_2_ will not transform into TiO_2_ under the reaction conditions of preparing ZnIn_2_S_4_/Ti_3_C_2_. Ti-O is formed during the preparation of Ti_3_C_2_ due to the substitution of O or OH for Tx in Ti_3_C_2_Tx [28]. Consistent with the Ti 2p spectrum, the C1s peak at 281.6 eV was assigned to Ti-C, and its intensity increased with increasing Ti_3_C_2_ content (Figure 7). Moreover, compared with pure ZnIn_2_S_4_, the Zn 2p and In 3d peaks in the XPS spectra of ZnIn_2_S_4_/Ti_3_C_2_ shifted towards higher binding energies, while the S 2p peak shifted to lower binding energy. These changes suggest a strong electronic interaction between Ti_3_C_2_ and ZnIn_2_S_4_ (Figure 8, Figure 9 and Figure 10).

**Figure 11:**
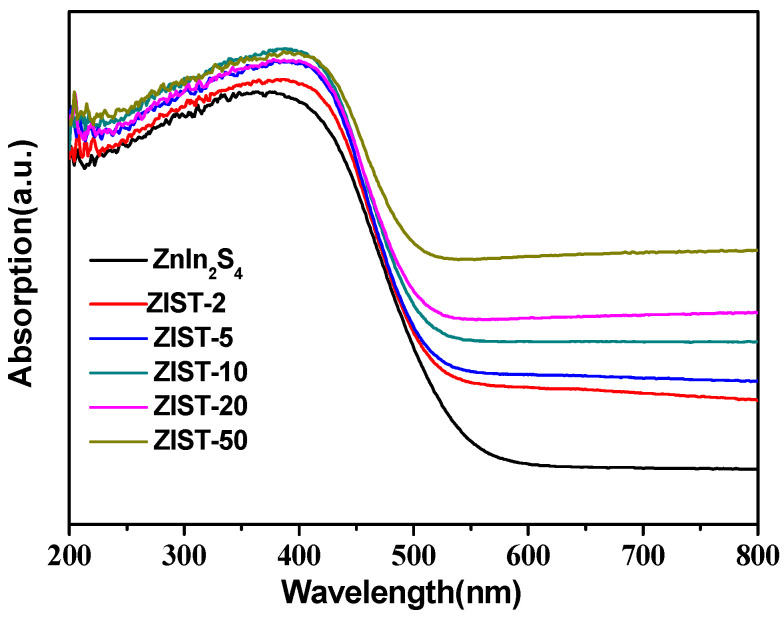
UV−vis DRS spectra of ZnIn_2_S_4_ and ZnIn_2_S_4_/Ti_3_C_2_ composites with different Ti_3_C_2_ content.

DRS was used to measure the light harvesting capability of the photocatalyst samples. A clear red shift was exhibited for the ZnIn_2_S_4_/Ti_3_C_2_ nanocomposites, and their absorption in the visible light region was significantly enhanced compared with pure ZnIn_2_S_4_ (Figure 11). As the Ti_3_C_2_ content increased, the absorption intensity of ZnIn_2_S_4_/Ti_3_C_2_ gradually increased due to the full-spectrum absorption of dark Ti_3_C_2_ [23]. Because of the high efficiency of photothermal conversion of Ti_3_C_2_ [22], it is possible to activate the catalyst by converting the light energy into heat to promote the surface catalytic reaction [29].

### 2.2. Photocatalytic Activity

**Figure 12:**
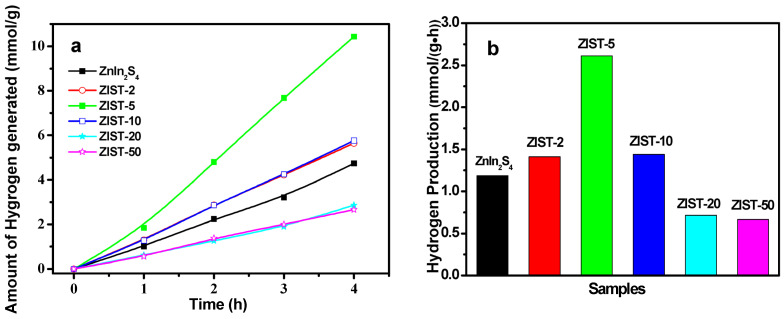
(**a**) Photocatalytic H_2_ evolution over ZnIn_2_S_4_ and ZnIn_2_S_4_/Ti_3_C_2_ composites with different Ti_3_C_2_ content under visible light irradiation. (**b**) Rate of H_2_ evolution of ZnIn_2_S_4_/Ti_3_C_2_ composites with different Ti_3_C_2_ content under visible light irradiation.

**Figure 13:**
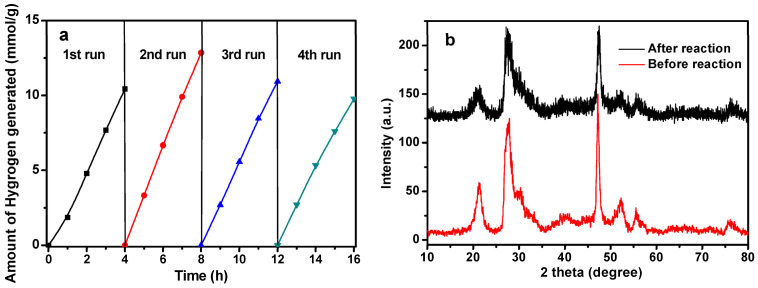
(**a**) Cycling tests of photocatalytic H_2_ production under visible light irradiation over ZIST-5. (**b**) XRD patterns for experimental ZIST-5 before and after the reaction.

**Figure 14:**
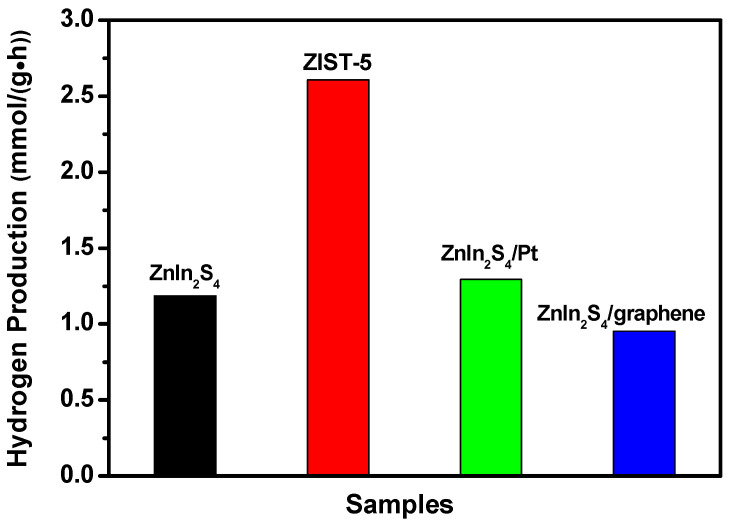
Rate of H_2_ evolution of ZnIn_2_S_4_, ZnIn_2_S_4_/Pt, ZnIn_2_S_4_/graphene, and ZIST-5 under visible light irradiation.

The photocatalytic performance of the samples was measured according to the hydrogen evolution from water under visible light irradiation (>420 nm). As expected, a significant enhancement in the photocatalytic activity for hydrogen evolution was observed upon loading of a small amount of Ti_3_C_2_ (Figure 12). When the mass ratio of Ti_3_C_2_ reaches 5% (ZIST-5), the best photocatalytic activity is obtained (2.6 mmol·g^−1^·h^−1^). However, with the further increase in Ti_3_C_2_ content, the photocatalytic activity gradually decreased. This may be due to an excess of Ti_3_C_2_ causing the composite materials to become too conductive. The result is the depression in charge separation efficiency over the composite [30]. In addition, the presence of Ti_3_C_2_ with a large amount of dark will also have a shading effect, which is not conducive to the light absorption of composite materials. In addition, the recyclability of ZIST-5 for photocatalytic hydrogen production was also evaluated. The cycling experiments show that the yield of hydrogen could still reach 2.4 mmol·g^−1^·h^−1^ after four cycles (Figure 13a). The XRD spectra show that the crystal structure of the recovered catalyst remains intact except for some weakening of crystallinity, indicating that it has excellent stability. For a comparison, the same proportions of Pt and graphene (5 wt.%) were loaded on ZnIn_2_S_4_, respectively. Under the same experimental conditions, ZnIn_2_S_4_/Pt and ZnIn_2_S_4_/graphene showed lower photocatalytic activities of 1.3 mmol·g^−1^·h^−1^ and 1.0 mmol·g^−1^·h^−1^ than of ZIST-5, respectively (Figure 14). These results indicate that ZIST-5 is a promising photocatalyst for photocatalytic hydrogen production.

## 3. Discussion

### 3.1. The Photoluminescence Spectra and Electrochemical Impedance Spectra

**Figure 15:**
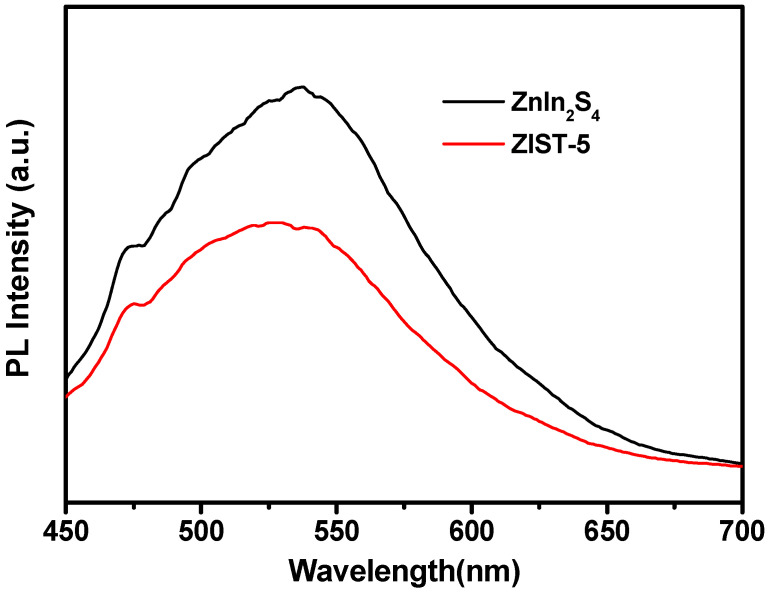
PL spectra of ZnIn_2_S_4_ and ZIST-5.

**Figure 16:**
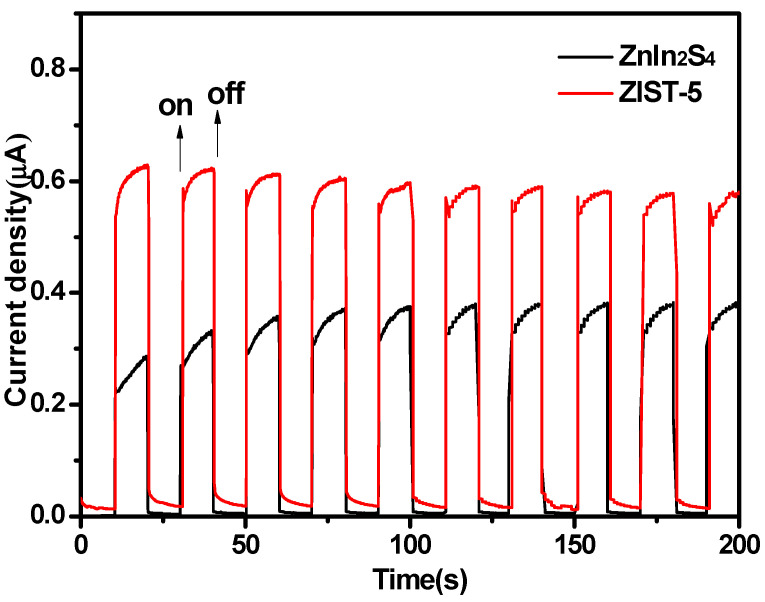
Transient photocurrent responses of ZnIn_2_S_4_ and ZIST-5 (300 W Xe lamp equipped with a 420 nm cut-off filter as a light source).

**Figure 17:**
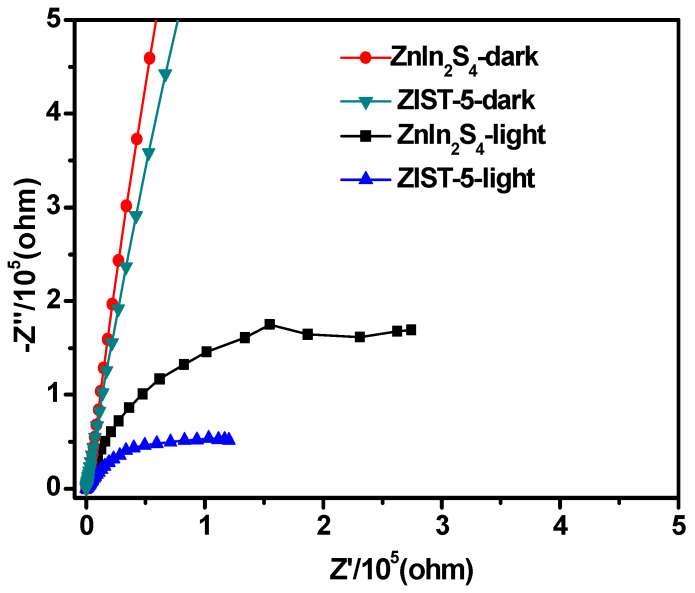
Nyquist plots of experimental impedance data for ZnIn_2_S_4_ and ZIST-5 in the frequency range of 4 MHz to 10 MHz using an AC bias of 0 V vs. Ag/AgCl without or with visible light irradiation (λ > 420 nm).

In order to further comprehend the role of Ti_3_C_2_ in improving the photocatalytic activity of ZnIn_2_S_4_, the photoluminescence (PL) spectra and electrochemical impedance spectra (EIS) were recorded. PL emission is generated by the recombination of electrons and holes; therefore, lower PL intensity indicates higher separation efficiency of photo-generated electron–hole pairs [30]. As shown in Figure 15, the PL spectra show that the main emission peaks of ZnIn_2_S_4_ and ZIST-5 appear at 537 nm, while the intensity of the emission peak of ZIST-5 is much weaker than that of ZnIn_2_S_4_. This suggests the heterogeneous junction formed between Ti_3_C_2_ and ZnIn_2_S_4_ can enhance the efficiency of photogenerated electron and hole separation. In photocurrent response spectra (Figure 16), ZIST-5 showed a higher photocurrent than ZnIn_2_S_4_ and, thus, more efficient separation and transportation of photoinduced charge carriers [31]. In the EIS Nyquist plot, a smaller arc radius indicates lower charge transfer resistance at the electrode surface and higher separation efficiency for electron–hole pairs [32]. In Figure 17, the arc radius of ZIST-5 is a little less than that of pure ZnIn_2_S_4_ in the darkness, suggesting that ZIST-5 has more effective separation of photogenerated charges. Under visible light irradiation, the arc radius of ZIST-5 and ZnIn_2_S_4_ significantly decreases compared with that of darkness, and ZIST-5 decreases more than that of ZnIn_2_S_4_. These results show that in ZIST-5, Ti_3_C_2_ acts as an electron acceptor to capture photoelectrons produced by ZnIn_2_S_4_ due to its good electrical conductivity. Moreover, it can increase the photocatalytic activity by effectively inhibiting photoinduced carrier recombination.

### 3.2. The Photocatalytic Mechanism

**Scheme 1:**
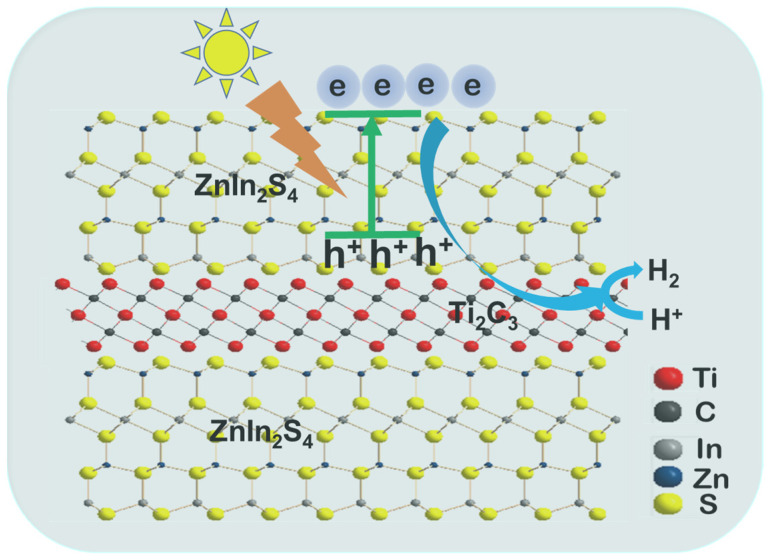
Suggested mechanism for the photocatalysis of Ti_2_C_3_/ZnIn_2_S_4_ under visible light.

Based on the findings presented above, we propose the possible mechanism of the photocatalytic reaction for this system as shown in Scheme 1. Upon visible light irradiation, electrons (e^−^) are excited from the valence band (VB) to the conduction band (CB) of the ZnIn_2_S_4_ semiconductor, creating holes (h^+^) in the VB. Electrons are transferred to the Ti_3_C_2_ nanosheets and accumulate. These electrons can effectively reduce H_2_O (or H^+^) to produce H_2_, and the accumulated (h^+^) in the VB of ZnIn_2_S_4_ react with S^2−^/SO_3_^2−^ as a regenerative reagent. The presence of Ti_3_C_2_ can serve as an excellent electron acceptor and mediator, with effective separation of holes and electrons. This results in a significant improvement in the photocatalytic activity.

## 4. Materials and Methods

### 4.1. Synthesis of Samples

All reagents were of analytical grade and used without further purification. Ti_3_AlC_2_ powder (>98 wt.% purity) was purchased from Beijing Lianli New Technology Co., Ltd., Beijing, China. By etching the Al layer of Ti_3_AlC_2_ with HF solution, Ti_3_C_2_ was obtained. In brief, 1 g Ti_3_AlC_2_ powder was slowly combined with 30 mL HF solution (content ≥ 40 wt.%, Xilong Chemical Co., Ltd., Shantou, China), and the mixture was stirred at room temperature for 72 h. The suspension was filtered and washed with deionized water several times, until neutral pH was achieved. The obtained Ti_3_C_2_ was dried under vacuum at 60 °C for 2 h.

Next, a hydrothermal reaction was employed to prepare the ZnIn_2_S_4_/Ti_3_C_2_ nanocomposites. An appropriate amount of Ti_3_C_2_ was dispersed in 70 mL of water by ultrasonication. To the Ti_3_C_2_ dispersion, ZnCl_2_ (0.136 g, 1 mmol, Tianjin Fengchuan Chemical Reagent Technology Co. Ltd., Tianjing, China) and excess L-cysteine were added, and the mixture was ultrasonicated. Subsequently, InCl_3_·4H_2_O (0.586 g, 2 mmol., Adamas Reagent Co. Ltd., Beijing, China) and thioacetamide (TAA, 0.300 g, 4 mmol., Sinopharm Chemical Reagent Co., Ltd., Shanghai, China) were added, and the solution was transferred to a 100 mL Teflon liner, sealed in a stainless steel autoclave, and heated at 150 °C for 5 h. The product was collected by centrifugation, washed several times with de-ionized water, and dried under vacuum at 60 °C for 12 h. The mass ratio of ZnIn_2_S_4_ to Ti_3_C_2_ was varied to determine the optimal composition, and mass ratios of 2 wt.%, 5 wt.%, 10 wt.%, 20 wt.%, and 50 wt.% corresponded to samples ZIST-2, ZIST-5, ZIST-10, ZIST-20, and ZIST-50, respectively.

### 4.2. Characterizations

Powder X-ray diffraction (XRD) data were collected using a Rigaku TTRAX III X-ray diffractometer (Rigaku D/max-3B, Tokyo, Japan) with Cu Kα radiation. A field emission scanning electron microscope (SEM) (FEIQuanta200FEG microscope, FEI, Eindhoven, The Netherlands) was used to examine the morphology of the sample. Transmission electron microscopy (TEM) and high-resolution transmission electron microscopy (HRTEM) images were obtained on a JEM-2100 microscope (JEOL, Tokyo, Japan). X-ray photoelectron spectroscopy (XPS) was performed on a K-Alpha XPS system (Thermo Fisher Scientific, Waltham, MA, USA) with a monochromatic Al Kα source. A Shimadzu UV-2600 photometer was used to record the UV–vis diffuse reflectance spectra (UV-vis DRS, Shimadzu, Kyoto, Japan), using BaSO_4_ as a reflectance standard. A PGSTAT 302N electrochemical analyzer (Metrohm, Herisau, Switzerland) was used to record the photocurrent response spectra and electrochemical impedance spectra (EIS) in a standard three-electrode system. The prepared samples were used as the working electrodes with an active area of ca. 0.25 cm^2^ on ITO conductive glass. Pt wire was the counter electrode, a standard calomel electrode was used as the reference electrode, and 0.5 M Na_2_SO_4_ aqueous solution was used as the electrolyte. A F-7000 fluorescence spectrophotometer (Hitachi, Tokyo, Japan) was used to record the photoluminescence (PL) spectra of powder samples at an excitation wavelength of 325 nm.

### 4.3. Photocatalytic H_2_ Generation

Photocatalytic H_2_ evolution experiments were conducted in a closed gas circulation and evacuation system fitted with a Pyrex glass window (LABSOLAR-H2I, Beijing Perfectlight Technology Co., Ltd., Beijing, China). In 100 mL of a mixed aqueous solution containing 0.05 M Na_2_SO_3_ (Xilong Scientific Co., Ltd., Shantou, China) and 0.05 M Na_2_S (Xilong Scientific Co., Ltd., Shantou, China), 20 mg of photocatalyst was suspended, and the suspension was irradiated with a 300 W Xe lamp (CEL-HXF300, China Education Au-light Co. Ltd., Beijing, China) equipped with a 420 nm cut-off filter. Using a circulating water bath, the solution was maintained at room temperature during the reaction. The concentration of H_2_ was directly detected by an on-line gas chromatograph (GC-9750, Chengdu Fuli Instrument Co., Ltd., Chengdu, China) equipped with a thermal conductivity detector (TCD).

## 5. Conclusions

In conclusion, a facile hydrothermal method was employed for the successful preparation of 2D/2D ZnIn_2_S_4_/Ti_3_C_2_ composite photocatalysts. The 2D/2D ZnIn_2_S_4_/Ti_3_C_2_ heterostructures showed excellent interface activity, ideal photoresponsivity properties, and high photogenerated carrier mobility. Our findings suggest Ti_3_C_2_ MXene has a unique synergistic effect on photocatalytic hydrogen production due to its unique two-dimensional structure, fast carrier migration rate, and ideal interfacial effect. The separation of photogenerated carriers and the transfer of photoelectrons to MXene nanosheets were significantly promoted by the incorporation of the heterojunction between ZnIn_2_S_4_ and Ti_3_C_2_. As a result, we observed a significant improvement in the photocatalytic activity. This work provides a novel synthetic method for the preparation of 2D MXene composite photocatalytic materials and expands the utility of these materials to energy storage and conversion applications.

## Data Availability

Data available on request.
